# Metagenome-Assembled Genomes for “*Candidatus* Phormidium sp. Strain AB48” and Co-occurring Microorganisms from an Industrial Photobioreactor Environment

**DOI:** 10.1128/mra.00447-22

**Published:** 2022-11-21

**Authors:** Avery J. C. Noonan, Yilin Qiu, Brandon Kieft, Sean Formby, Tony Liu, Kalen Dofher, Moritz Koch, Steven J. Hallam

**Affiliations:** a Genome Science and Technology Program, University of British Columbia, Vancouver, British Columbia, Canada; b ECOSCOPE Training Program, University of British Columbia, Vancouver, British Columbia, Canada; c Department of Microbiology and Immunology, University of British Columbia, Vancouver, British Columbia, Canada; d Graduate Program in Bioinformatics, University of British Columbia, Vancouver, British Columbia, Canada; e Life Sciences Institute, University of British Columbia, Vancouver, British Columbia, Canada; Indiana University, Bloomington

## Abstract

Here, we report metagenome-assembled genomes for “*Candidatus* Phormidium sp. strain AB48” and three cooccurring microorganisms from a biofilm-forming industrial photobioreactor environment, using the PacBio sequencing platform. Several mobile genetic elements, including a double-stranded DNA phage and plasmids, were also recovered, with the potential to mediate gene transfer within the biofilm community.

## ANNOUNCEMENT

Cyanobacteria offer the possibility of producing energy and materials from sunlight and carbon dioxide (1, 2). However, these applications of cyanobacteria remain limited, and more industrial strains with desirable growth properties are needed (1, 3, 4). Recent studies indicate that members of the *Phormidium* genus have potential to fill this gap (5–7). The genus represents a polyphyletic distribution of filamentous cyanobacteria containing over 200 species, many of which form dense biofilms with co-occurring microorganisms (8–10).

Here, we describe metagenome-assembled genome (MAG) sequences, including mobile genetic elements (MGEs), from an industrial photobioreactor environment. The photobioreactor uses high temperature (35 to 45°C), salinity (10 g/L), and alkaline conditions (pH 9 to 11) to support biofilm-based growth. After several months of continuous cultivation, with harvesting every 48 to 96 h, a biofilm sample was collected. Genomic DNA was extracted from biofilm biomass using a cetyltrimethylammonium bromide (CTAB)-chloroform extraction protocol (11). DNA was sheared to 10 kb using Covaris g-TUBES and size selected (>10 kb) with AMPure beads (Beckman Coulter). This high-molecular-weight fraction was used to prepare a Pacific Biosciences (PacBio) SMRTbell library for sequencing on the PacBio Sequel platform. Default parameters were used for sequencing data processing and analysis software, unless otherwise noted. A total of 1,551,061 reads (5,417 Mbp) were filtered and trimmed using BBTools (v.38.79) (parameters: jni=t json=t ow=t cq=f keepshortreads=f trim=f), resulting in 1,417,931 reads (4,615 Mbp) with an *N*_50_ value of 5,590 bp. The metagenome was assembled with Flye (v.2.7) using meta parameters, and the Flye assembly graph was converted into FASTA format for downstream processing (12). Contigs of >1,000 bp were assigned to population genome bins using MaxBin2 (v.2.2.6) (13), and the completeness and contamination of resulting MAGs were assessed using CheckM (v.1.1.3) (14).

A total of 281 contigs, with an *N*_50_ value of 56,161 bp and a total length of 13,019,354 bp as evaluated by QUAST (v.5.0.2) (15), were assembled. Four MAGs were resolved, including a complete genome and three additional population genome bins of varying quality. The complete genome was 4,818,683 bp, with a GC content of 51.64% and an *N*_50_ value of 4,751,363 bp. A high-quality genome of 3,207,041 bp, with CheckM completeness of 89.03%, a GC content of 49.23%, and an *N*_50_ value of 51,694 bp, and two draft-quality genomes of 3,511,511 bp and 1,359,261 bp, with completeness values of 37.80% and 23.31%, GC contents of 59.57% and 63.91%, and *N*_50_ values of 30,355 bp and 25,610 bp, respectively, were identified. MGEs, including putative phages and plasmids, were identified using PlasFlow (v.1.1), VirSorter2 (v.2.2.2), and CheckV (v.0.8.1) (16–18). A total of three circular plasmids, one high-quality double-stranded DNA (dsDNA) phage (100% completeness), and one medium-quality dsDNA phage (50.59% completeness) were predicted. One of the plasmids, 51,598 bp in length, clustered with the complete cyanobacterial MAG. The remaining contigs passing the quality control (QC) threshold (0.94% of total bases) could not be assigned.

Analysis of single-copy marker genes in the four MAGs using GTDB-Tk (v.0.3.2) (19) and an average nucleotide identity (ANI) of 87.75% with its closest relative, *Phormidium_A* sp007126595 (GCA_007131565.1), as calculated using fastANI (v.1.1) (20), indicated that the complete genome represents a novel member of the cyanobacterial *Phormidium_A* genus, designated “*Candidatus* Phormidium sp. strain AB48” (21). The high-quality genome was assigned to the *Alteromonadaceae* family, and the two draft-quality genomes were assigned to the *Verruco 01* and *Maricaulaceae* families. Open reading frame (ORF) prediction and genome annotations were performed using Prokka (v.1.14.5) (22). The complete “*Candidatus* Phormidium sp. strain AB48” genome contains 4,372 genes, 4,318 coding sequences (CDSs), 6 rRNA genes (two 5S rRNAs, two 16S rRNAs, and two 23S rRNAs), and 47 tRNAs.

### Data availability.

Data from this project are publicly accessible through the NCBI under BioProject accession no. PRJNA834472 and BioSample accession no. SAMN28044958. Raw sequencing data are available from the Sequence Read Archive (SRA) under the SRA accession no. SRR13132258. This BioProject also includes BioSample SAMN28044976; this represents a distinct sample and contains Oxford Nanopore Technologies and Illumina data sets for Phormidium yuhuli AB48, which was isolated from the photobioreactor enrichment described here (23).
